# Infantile Colic and Long-Term Outcomes in Childhood: A Narrative Synthesis of the Evidence

**DOI:** 10.3390/nu15030615

**Published:** 2023-01-25

**Authors:** Flavia Indrio, Vanessa Nadia Dargenio, Ruggiero Francavilla, Hania Szajewska, Yvan Vandenplas

**Affiliations:** 1 Department of Medical and Surgical Science Pediatric Section, University of Foggia, 71100 Foggia, Italy; 2 Interdisciplinary Department of Medicine, Pediatric Section, Children’s Hospital ‘Giovanni XXIII’, University of Bari Aldo Moro, 70126 Bari, Italy; 3 Department of Pediatrics, The Medical University of Warsaw, 02-091 Warszawa, Poland; 4 UZ Brussel, KidZ Health Castle, Vrije Universiteit Brussel, 1090 Brussels, Belgium

**Keywords:** infantile colic, migraine, atopic eczema, food allergy, behavioral disorders, developmental disorders

## Abstract

About 1 in 4 infants comes forward with prolonged crying, agitation, or infant colic (IC) during the first three months of life and is referred for medical evaluation. The pathogenesis remains poorly understood, as do its implications for future health. The aim of this narrative review was to critically examine and discuss the available literature on long-term consequences of excessive crying and/or colic. Most studies display an association between IC and the onset of functional gastrointestinal disorders (FGIDs) years later, probably related to the presence of common etiopathogenetic factors (environmental, dietary, intestinal dysmotility, visceral hypersensitivity). Although allergic disease in first-degree relatives may be a risk factor for IC, the latter does not appear to be a risk factor for subsequent atopic disease in the individual. Overall, there seems to be a relationship between IC and subsequent headaches, of the migraine type. Similarly, behavioral problems in children with a history of IC appear to be associated with higher parental stress scores. However, the current evidence is based on associations, and currently, a causal relationship between excessive crying and IC and long-term consequences remains not documented.

## 1. Introduction

Infant colic (IC) is commonly observed in the first three months of childhood and affects 2% to 73% of newborns worldwide, depending on the diagnostic criteria used [1,2]. IC generally resolves in the first six months of life, but persistent symptoms can contribute to parental anxiety, causing repeated reports to physicians [3]. Their pathogenesis remains poorly understood, as do their implications for future health. This condition is described as a multifactorial syndrome [4]. A pathophysiological role in the development of IC may be played by the complex interactions between behavioral factors (psychological and social), nutritional factors (hypersensitivity or food allergies), an immaturity of the structure and function of the gastrointestinal tract, an excessive amount of gas in the gut, abnormalities of intestinal hormones, and gut dysmotility [5,6,7]. One of the most recent theories assigns a key role to the imbalance in the composition of the intestinal microbiota that would seem to affect the occurrence of IC [8]. An immaturity of the microbiota and nervous and immune systems of the gastrointestinal tract exists at birth [9]. The appropriate development of the gut microbiota, which is essential for proper maturation of the immune system and has beneficial effects on the whole body, is strongly influenced by the neonatal period [10]. Reduced microbial diversity, elevated levels of calprotectin, and antimicrobial proteins released by intestinal neutrophils probably related to low-grade intestinal inflammation have been identified in colicky infants compared with healthy infants [11]. Other recent hypotheses suggest the possible role of postpartum depression, parental anxiety, stressful pregnancies, adverse experiences during childbirth, poor parenting skills, and dysregulation of the gut–brain axis in the etiopathogenesis of IC.

In this context, it is interesting to note that several sequelae seem to be associated with the presence of IC early in life, such as gastrointestinal disorders, psychological and allergic disorders, such as atopic eczema and food allergy, migraine, and behavioral and developmental disorders [12,13,14,15].

If IC is an early manifestation of these disorders, this would have significant implications for their treatment and prevention.

The purpose of this narrative review was to critically explore and discuss the existing published literature on long-term consequences of excessive crying and/or colic. In addition, based on the evidence from the relevant available literature, it is intended to establish whether there is an association between excessive crying time and/or colic in early childhood and the long-term health implications for infants.

## 2. Materials and Methods

Two independent authors (VD and FI) searched the PubMed, Embase, and Web of Science databases for articles published from inception through 01 August 2022 on the long-term consequences of excessive crying and/or colic.

The following search keywords were used in the databases: IC, functional gastrointestinal disorders (FGIDs), migraine, headache, allergic disorders, asthma, atopy, behavior, sleep mood, hyperactivity, attention-deficit/hyperactivity disorder (ADHD), autism spectrum disorder (ASD), cognitive development, colic symptoms, daily crying, as well as their combinations.

Observational studies (retrospective, cross-sectional, or prospective) and randomized clinical trials that discussed the relationship between excessive crying and/or colic and their long-term consequences were included. The literature search was limited to articles published in English in peer-reviewed journals in order to select studies that underwent a rigorous international peer-review process.

## 3. Search Results

Among the articles included there were 5 articles concerning the correlation between IC and onset of FGIDs; 4 articles on allergy-based disorders; 3 on the risk of migraine onset; and 10 on alterations in behavior, sleep mood, hyperactivity, ADHD, ASD, and cognitive development.

Table 1 provides a summary of the studies included in the results.

## 4. Discussion

### 4.1. Functional Gastrointestinal Disorders

FGIDs have been defined as a variable combination of chronic or recurrent gastrointestinal symptoms unrelated to organic pathology. A 2015 study estimated the worldwide prevalence of FGIDs-related abdominal pain to be 13.5%, and the most common disorder was found to be irritable bowel syndrome [28].

FGIDs compromise the quality of life of children as they are associated with significant morbidity and require a high cost to health care.

Their etiopathogenesis has not yet been clarified, and there is no diagnostic test that leads to a definitive diagnosis. However, some etiological factors have been identified, such as visceral hypersensitivity, infections, genetic factors, stress, and alterations in the gut microbiota. Evidence suggests that there is a constant bidirectional dialogue between brain and gut, which seems to be the mechanism linking emotional state with gastrointestinal dysfunction. Finally, recent preclinical studies indicate that changes in the gut microbiota may influence brain signaling systems related to pain and its associated emotional behavior. Traditionally, they have been considered self-limited diseases; however, altered microbiota and trans-mucosal passage of antigens across the immature intestinal barrier may play a crucial role in the pathogenesis of these functional disorders [29]. Furthermore, it has been hypothesized that this kind of early traumatic insult could be a risk factor for the onset of other FGIDs such as recurrent abdominal pain later in childhood [30].

Based on these hypotheses, several researchers have attempted to study the long-term impact of FGIDs in infants not only to identify its pathogenesis but also to seek an effective treatment or preventive approach.

Indrio et al. conducted a large retrospective study of patients with recurrent abdominal pain that were referred to a pediatric gastroenterology outpatient clinic from January 2002 to December 2009 [16]. The diagnosis of FGIDs related to abdominal pain was made according to Rome II or Rome III diagnostic criteria valid at the time of evaluation. In total, 2987 patients (1875 males, mean age 7.9 years) were enrolled. The study population was matched with 3121 healthy controls (1789 males, median age 7.5 years), age- and gender-matched, with no history of recurrent abdominal pain enrolled among pediatricians practicing primary health care. History of IC, regurgitation, and functional constipation was found in 26.41, 25.31, and 30.16 percent of children diagnosed with FGIDs compared with 11.34, 12.85, and 11.76 percent of healthy children, respectively (*p <* 0.001, odds ratio (OR) 2.81; *p <* 0.001, OR 2.30; and *p <* 0.001, OR 3.24).

Despite the limitations of the study, based on these results obtained from a large population, it can be hypothesized how the early occurrence of bowel discomfort may be a risk factor for the development of recurrent abdominal pain.

These hypotheses were subsequently confirmed and extended by further studies.

A prospective survey with follow-up at 4 years of age on FGIDs conducted on 50 formerly colicky children and 102 healthy controls was conducted in Sweden [19]. Formerly colicky children differed significantly from controls with regard to complaining about stomach-ache (*p* = 0.037), while complaining about aches “in other parts of body” was somewhat more frequent in healthy subjects, although there was no statistically significant difference (*p* = 0.08). Similarly soiling and constipation were more common in the Control Group but not statistically significant (*p* = 0.053 and 0.097, respectively).

Similarly, Savino et al. conducted a prospective study of 96 infants who were enrolled from January 1991 to December 1993, including 48 infants who had been examined for severe IC and 48 controls [18]. At age 10 years, each group was subsequently reevaluated for gastrointestinal complaints (gastritis and peptic ulcer, recurrent abdominal pain and constipation). Recurrent abdominal pain was observed in 33.33% of ex-colic infants and 4.42% of ex-non-colic infants (*p* = 0.001; RR 10.7). No significant difference between the two groups of infants was found for the other gastrointestinal disorders examined. The results of this study show an association between IC and recurrent abdominal pain, in agreement with what has been shown in previous studies listed above.

Partty et al. conducted a randomized, double-blind, prospective follow-up study of probiotic intervention (*Lacticaseibacillus rhamnosus* GG (ATCC 53103; Valio Ltd. Helsinky, Finland) or placebo) to assess the risk of occurrence of FGIDs later in life [17]. The preparation was administered to 159 mothers for 4 weeks before delivery, then given to the infant or continuously to the mother, if breastfeeding, for 6 months. At 13 years of age, the subjects (n = 75) were followed up, and the diagnosis of FGIDs was based on the modified Rome III Diagnostic Questionnaire for Pediatric FGIDs Diagnosis [31].

The diagnosis of FGIDs was made in 15 children (20.3%), of whom 28% and 5.6% of children with and without colic-like crying during the seventh week of life, respectively, developed FGIDs (*p* = 0.05). Likewise, children manifesting with FGIDs had more colic-type crying than healthy children during the seventh week of life (*p* = 0.005). The same tendency was still observed during the 12th week of life (*p* = 0.08). Other modes of infant distress were comparable between the groups (Abdominal migraine and IBS, being the most common FGIDs diagnoses). Early probiotic supplementation had no effect on the development of FGIDs, suggesting that different probiotics or combinations of probiotics may be more effective in the treatment of pediatric FGIDs.

The results obtained from these studies should not be surprising, as the etiopathogenetic factors (environmental factors, dietary factors, intestinal dysmotility, visceral hypersensitivity) and symptoms (increased intraluminal gas, irritability) are common to both IC and FGIDs [32]. Indeed, it is well known that gastrointestinal dysmotility is a characteristic of infants with colic but also of individuals with recurrent abdominal pain [33,34]. At the intestinal level is located one of the largest lymphoid organs in the human organism: inflammatory cytokines and chemokines, mast cell degranulation, and their interaction with the enteric nervous system are among the possible etiopathogenetic factors of gastrointestinal hypermotility and consequently cause the development of recurrent abdominal pain [34].

In another study, not only the evidence published in the literature but also the opinions of practicing physicians from around the world and international experts on the prevalence and long-term health consequences of FGIDs in infants younger than 12 months were reviewed [2]. As is already known, the consensus concludes that the worldwide prevalence of IC is uncertain, estimated at about 20 percent, and may have a long-term impact on future health outcomes in a subset of children.

### 4.2. Allergy and Atopic Diseases

Cow’s milk elimination diets with extensively hydrolyzed formula have been reported as effective in IC [35,36]. Since allergic disease acquired early in life is considered as a possible risk factor for the onset of the atopic march, predisposing for later allergic disease, the question arises if IC predisposes for the onset of allergic disease later in life. Savino and coworkers reported that a family history of gastrointestinal diseases ad atopic diseases was significantly higher in infants with colic than in controls (*p* < 0.05) [18].

Savino and colleagues re-evaluated in a prospectively designed study 103 infants with IC at the age of 10 years [18]. In total, 96/103 completed the study, and an association was found between IC and recurrent abdominal pain (*p* = 0.001) and allergic disorders (allergic rhinitis, conjunctivitis, asthmatic bronchitis, pollenosis, atopic eczema and food allergy) (*p* < 0.05) [18]. Cerrato et al. reported a post hoc analysis of 50 breastfed infants with IC [28]. In a post hoc follow up of 48 of these 50 children when 4 years old (25 treated during infancy with *Limosilactobacillus* (*L*.) *reuteri* DSM 17938 for IC and 23 children treated with placebo), no difference in asthma (0 in both groups), in migraine (1 in the placebo group vs. none on the *L. reuteri* group), but a significantly decreased prevalence of atopic disorders (8 (34.8%) in the placebo group vs. 2 (8%) (OR 0.16; 95% CI 0.03-0.88) in the *L. reuteri* group at five years of age) [20]. In a prospective follow-up study 116 newborns with at least one first-line relative with atopic eczema, allergic rhinitis or asthma were followed up to the age of 2 years [14]. Atopic disease was diagnosed at age 2 years in 38% (44/116) and asthma in 5% (6/116) [14]. Crying at the 7th week of life was not different in the group with or without allergy at 2 years of age but fussing (*p* = 0.02) was. At the 12th week of life, colic was more frequent in the allergic group (*p* = 0.04) and overall distress was not [14]. Castro-Rodriguez et al. reported in a series of 90 infants with IC, with a similar prevalence in breastfed (9.2 % out of 425 infants) and formula fed (7.6% out of 316 infants), the absence of an association between IC and markers of atopy, asthma, allergic rhinitis, wheezing or peak flow variability at any age [21]. Children were followed up to the age of 14 years [21].

### 4.3. Migraine

A 2015 meta-analysis [26] of three studies, including two case–control studies [4,37] and one cross-sectional study [38], showed that compared with infants without IC, those with IC had increased odds of migraine (n = 891, OR 6.5; 95% CI 4.6-8.9, *p* < 0.001, for the fixed-effects model and OR 5.6; 95% 3.3-9.5, *p* = 0.004, for the random-effects model). In a sensitivity analysis, after excluding the study with the largest effect size [4], increased odds remained for both the fixed-effects and random-effects models (OR 3.6, 95% CI 1.7–7.6, *p* = 0.001). Included studies were prone to recall bias, so caution is needed when interpreting the findings.

One subsequently published prospective cohort study involved 1107 Finish infants with IC diagnosed until the age of 3 months who were followed-up at ages 3, 12, 15 and 18 years (n = 787). Migraine was present in 22/96 (23%) adolescents with a history of IC compared with 74/658 (11%) adolescents with no such history. Among 22 adolescents with a migraine, significantly more subjects had a migraine without aura than with aura (14/22 (64%) vs. 8/22 (36%) [27].

Overall, the findings suggest that IC is associated with an increased risk of migraine, compared with no IC. Future studies on the mechanisms behind the associations are justified.

### 4.4. Behavioral Problems

A 2011 systematic review and meta-analysis assessed the associations between problems with crying, sleeping and/or feeding in infancy and long-term behavioral outcomes in childhood. Out of 22 included studies (search date: 2006), 10 reported on the consequences of excessive crying (defined as crying with intense, unshootable crying bouts for no apparent reason in the first 3 months of life) [18,19,22,23,25,39,40,41,42,43]. In the first six studies, these were transient problems. In the remaining four studies, there were persistent regulatory problems (defined as excessive crying beyond the third month of life and sleeping and feeding problems that occurred at initial assessment and at follow-up). Overall, this systematic review concluded that there was a strong association between crying problems during infancy and behavioral problems during childhood such as externalizing problems and ADHD. Heterogeneity of the studies, including the heterogeneity of the outcomes and methods of their assessment, and the small sample size in some of the included studies, indicate that further studies are needed. Below some of the included studies and subsequently published studies are discussed in more detail.

In 1995, a Finish prospective study reported data on 865 (72%) infants with IC (diagnosed by mothers) out of 1204 initially enrolled. At a 3-year follow-up, infants with IC had more sleeping problems and more frequent temper tantrums as measured with questionnaires compared with children who had no IC during infancy. Moreover, the families of the parents of infants who had a history of IC more often reported unsatisfaction with the arrangement of daily family responsibilities and decreased shared leisure time compared with families of children who had no IC [22].

In 2000, a Swedish prospective 4-year follow-up study of 50 infants with IC (however, defined according to four different definitions) and 102 control children found no differences were found in the two groups regarding most everyday behavioral aspects. The only exception was “temper tantrums” (based on a temperament questionnaire) that were more common in the ex-colicky group (*p* = 0.007) [19].

In 2005, an Italian prospective study of 96 infants found that those who had a history of IC (n = 48) compared with those with no history of IC (n = 48) at 10 years of age were more aggressive (41.67% vs. 6.20%, respectively), had more feelings of supremacy (37.5% vs. 4.42%, respectively), and were fussier (68.65% vs. 14.58%, respectively). No differences were found for enuresis or enjoying nursery/school [18].

In 2017, a Dutch population-based multiethnic birth cohort study (n = 3369) assessed the associations between an excessive infant crying (crying for 3 h per day for every day in one week) during the 13th week after birth, maternal burden of infant care, and maternal aggressive behavior (either angry speaking, or physical aggression) using a questionnaire. The study found that excessive crying was associated with an increased risk of maternally reported conduct problems, hyperactivity, mood problems, and overall problem behavior at the age of 5–6 years [ORs between 1.75 (95 % CI 1.09–2.81) and 2.12 (95 % CI 1.30–3.46)]. This association was mediated by maternal burden of infant care (change in odds’ ratio 1–17 %) and maternal aggressive behavior (change in odds’ ratio 4–10 %) [24].

Finally, in 2022, a prospective cohort study of Dutch infants with IC (according to the Wessel criteria; n = 258) had increased odds of scoring in the clinical range of the emotionally reactive (adj OR 2.96, 95% CI 1.24–7.06], internalizing (adj OR 2.50, 95% CI 1.35–4.62]; and total problems scale (adj OR 2.98, 95% CI 1.46–6.07). Internalizing (*p* < 0.001), externalizing (*p* < 0.001), and total (*p* < 0.001) behavioral problems in children with a history of IC were associated with higher parenting stress scores [44].

### 4.5. Intelligence

A 2004 US prospective cohort study with a 5-year follow-up found that children with prolonged excessive crying (n = 15) had an increased risk of lower IQ scores compared with the control group (n = 264) (adj. difference −9.2, 95% CI −16.5 to −2.0), hyperactivity, and discipline problems. No such problems were found in those with IC only (n = 48). IC had no effect on cognitive development [43]. The small number of children with prolonged crying and colic is one of the limitations of the study.

## 5. Conclusions

To our knowledge, the long-term outcomes of IC remain a very unexplored field, and more evidence is needed to draw strong conclusions.

Data obtained from the various studies seem to confirm the multifactorial etiology in IC since a high percentage of children with IC show allergic and psychological disorders later in childhood, so these factors then may be playing an important role in the etiopathogenesis. However, the current evidence is based on associations, and currently, a causal relationship between excessive crying and IC and long-term consequences remains undocumented.

Most studies show an association between IC and the occurrence of FGIDs, probably as a consequence of common etiopathogenetic factors (environmental, dietary, intestinal dysmotility, visceral hypersensitivity) and symptoms (increased intraluminal gas, irritability) as well. Although allergic disease in first-degree relatives may be a risk factor for IC, IC does not appear to be a risk factor for subsequent atopic disease in the individual. However, these results could be biased by the fact that more breastfed children were included. In addition, although somewhat contradictory regarding the association between IC and type of migraine or headache, overall, there seems to be a relationship between IC and the subsequent headache of the migraine type. Similarly, behavioral problems in children with a history of IC appear to be associated with higher parenting stress scores.

Finally, we emphasize the lack of universal definitions of excessive crying and/or IC in all included studies. Limitations of some studies include a rather small study population, a considerable dropout rate, heterogeneity of study designs, outcomes assessed and assessment methods, and the presence of significant independent confounding effects of genetic factors such as parental IQ or mode and duration of breastfeeding. New assessments such as children’s and parents’ quality of life, sleep time, and the impact of the condition on parents could probably be useful.

The collection of reliable data in prospective long-term follow-up studies according to agreed, more homogeneous, and well-defined criteria is needed to obtain more accurate estimations of long-term sequelae of these common childhood symptoms, to confirm these correlations, but also to find effective preventive approaches and new treatment options.

## Figures and Tables

**Table 1:** Summary of studies on long-term consequences of excessive crying and/or colic.

Reference	Study Type	Population	Results	Author’s Conclusion
Colic and FGIDs				
Indrio F et al. [16]	Retrospective	N = 2987 with FGIDs + N = 3121 healthy controls	History of IC was detected in 26.41% of children diagnosed with FGIDs compared to 11.34% of healthy controls (*p* < 0.001, OR 2.81)	Children with a history of IC have a higher prevalence of FGIDs years later
Partty A et al. [17]	Prospective, follow up study at 13 years	N = 75 (children participating in RCT of probiotic intervention (L. rhamnosus GG ATCC 5310) in perinatal period)	FGIDs were observed in 28% and 5.6% of children with and without colic-type crying during the seventh week of life, respectively (*p* = 0.05)	The early probiotic supplementation had no effect on the development of FGIDs
Savino F et al. [18]	Prospective, follow up study at 10 years	N = 96 (aged 10 years, 48 with history of IC + 48 healthy controls)	Recurrent abdominal pain was observed in 33.33% and 4.42% of infants with and without IC, respectively (*p* = 0.031)	Susceptibility to recurrent abdominal pain in childhood may be increased by IC
Vandenplas Y et al. [2]	Review, questionnaire to clinicians	N = 369 clinicians	Prevalence of IC is estimated to be approximately 20% and may have an impact on future health outcomes	Prospective studies are needed to obtain more accurate estimates of consequences of IC
Canivet C et al. [19]	Prospective, follow up study at 4 years	N = 50 with history of IC + N = 102 healthy controls	Stomach-aches were more common in ex-colicky children (*p* = 0.037)	There are no serious long-term complications of IC
Colic and allergic disorders				
Cerrato S et al. [20]	Longitudinal, follow up study at 5 years	N = 50 (25 colicky infants receiving L. reuteri DSM 17983 + 23 placebo)	Prevalence of atopic disorders was 8% in intervention group vs. 34.8% in placebo group at five years of age (OR 0.16; 95% CI 0.03–0.88)	The use of L. reuteri in the treatment of IC decreased prevalence of atopic disorders at five years of age
Kalliomaki M et al. [14]	Prospective	N = 116 high risk new-born infants for allergic disorders	IC was significantly greater in children developing atopic disease than in controls (*p* = 0.04)	Studies are needed to assess gut barrier functions and possible modulation of gut microflora to prevent allergy
Castro-Rodriguez JA et al. [21]	Prospective	N = 1246 (90 with IC)	9.2% of children had IC	There was no association between IC and markers of atopy, asthma, allergic rhinitis or wheezing at any age
Savino F et al. [18]	Prospective, follow up study at 10 years	N = 96 (aged 10 years, 48 infants who had been examined for severe IC + 48 controls)	There was an association between IC and allergic disorders (*p* < 0.05); no differences were found for anaphylaxis and urticaria	Susceptibility to allergic disorders in childhood may be increased by IC
Colic and behavior, sleep, hyperactivity, ADHD, ASD, cognitive development				
Canivet C et al. [19]	Prospective, follow up study at 4 years	N = 152 (50 with history of IC + 102 healthy controls)	No differences were found in the two groups regarding sleeping habits and behavior scales	There are no serious long-term complications of IC
Rautava P et al. [22]	Prospective, follow up study at 3 years	N = 1204 (338 with IC)	IC children had more sleeping problems and more frequent temper tantrums than the control group at 3 years of age	The families with IC infants had more distress 3 years later
Rao MR et al. [23]	Prospective, follow up study at 5 years	N = 327	Performance and verbal IQ scores of children with prolonged crying were 9.2 and 6.7 points lower than the control group, respectively (*p* < 0.05 and *p* = 0.06); they also had poorer fine motor abilities compared with the control group (*p*< 0.05)	IC had no effect on cognitive development
Smarius L et al. [24]	Prospective and observational	N = 3369 (102 excessive infant crying)	Excessive infant crying doubles the risk of behavioral, hyperactivity, and mood problems at the age of 5–6 [ORs between 1.75 (95 % CI 1.09–2.81) and 2.12 (95 % CI 1.30–3.46)]	Special care for mothers with a high care load for their baby who cries excessively may be a strategy to prevent behavior problems later in life
Savino F et al. [18]	Prospective, follow up study at 10 years	N = 96 (aged 10 years, 48 infants with a history of IC + 48 controls)	Sleep disorders were found in 56.25% of ex-colicky subjects and in 12.50% of ex-non-colicky infants, aggressiveness was found in 41.67% vs. 6.20%, a feeling of supremacy was found in 37.50% vs. 4.42%.	Susceptibility to psychological disorders in childhood may be increased by IC
Wolke D et al. [25]	Prospective, follow up study at 8–10 years	N = 64 persistent crying in infancy + N = 64 healthy controls	18.9% of children with persistent crying in infancy had pervasive hyperactivity problems vs. 18.9% of controls; academic achievement was reported by teachers to be significantly lower for children with persistent crying than controls	Children with persistent crying in infancy are at increased risk for hyperactivity problems and academic difficulties in childhood
Colic and Migraine				
Gelfand A et al. [26]	A systematic review and meta- analysis.	3 studies included in the primary analysis	OR for the association between migraine and infant colic was 6.5 (*p* < 0.001) for the fixed-effects model and 5.6 (*p* = 0.004) for the random-effects model	IC was associated with increased OR of migraine
Sillanpää M et al. [27]	Prospective, Follow-up study at ages 3, 12, 15 and 18 years	N = 787 (96 with history of IC)	23% of adolescents with history of IC suffered from migraine vs. 11% who had no history. Of the these, 64% had migraine without aura and 36% had migraine with aura.	Infants with IC had an almost three-fold risk for adolescent migraine without aura, but no increased risk for migraine with aura
Romanello S et al. [4]	case–control study	N = 679 (208 with migraine + 471 heathy controls)	72.6% of adolescents with history of IC suffered from migraine vs. 26.5% who had no history (*p* = 001), either migraine without aura (73.9% vs. 26.5%), or migraine with aura (69.7% vs. 26.5%)	Presence of migraine in children was associated with a history of IC.

N, number of patients; FGIDs, functional gastrointestinal disorders; IC, infantile colic; OR, odds ratio; ADHD, attention-deficit hyperactivity disorders; ASD, autism spectrum disorder.

## Data Availability

All data are presented within the manuscript text and tables.
